# Lectures on Clinical Surgery

**Published:** 1855-10

**Authors:** James Syme

**Affiliations:** Professor of Clinical Surgery in the University of Edinburgh


*Lectures on Clinical Surgery.* Delivered during the winter session of 1854–5. By James Syme, Esq., Professor of Clinical Surgery in the University of Edinburgh.

### LECTURE XX.—LITHOTOMY.

The next case to which I wish to direct your attention is that of Matthew W———, aged sixty-two, a weaver, from Dunfermline, who has suffered for the last fourteen years from urinary symptoms. He has been long in the practice of drawing off his urine by a catheter, on account of contraction in the urethra; yet remarkable as it may appear, he was not conscious of the presence of a stone in the bladder until I sounded him. On his admission, ten days ago, he was in a state of great irritability; his pulse being rapid, his tongue very dry, and his urine, which was excessively fetid, abounding in albumen, mucus, and pus; whilst he was in constant suffering night and day, and unable to use the catheter himself on account of nervous excitement. Since he has been under treatment here he has improved very considerably; his pulse is now of natural frequency; his tongue moister; the urine much improved, and his countenance wears occasionally a cheerful expression in the short intervals of ease which he now enjoys. Still he suffers very much from the presence of the stone, and as he is anxious for its removal, I intend to operate the day after to-morrow, and may therefore take this opportunity of making some remarks on the subject of lithotomy.

Before speaking of the operations, however, I have to say a single word regarding the symptoms of stone, which may be done very shortly, as they consist in reality chiefly of the evidence obtained by sounding. No symptoms, however striking, should ever be trusted without this process, and, on the other hand, though there be but slight symptoms of urinary irritation, you should not, therefore, conclude that stone is certainly absent. Still there are some symptoms which lead you more especially to suspect stone. If the patient has a frequent desire to pass urine from uneasines, which is relieved after micturition, then the case is probably one of mere irritability. If the pain occurs during micturition only, there is probably obstruction to the flow of urine, such as is produced by stricture; but if the pain be little or none at the beginning of micturition, increases towards the close of the act, and lasts with severity for a short time after it, then the probability is, that there is a stone in the bladder. There are also other symptoms of more or less importance; but without entering upon them, I must hasten to speak of the operation.

The stone is in this case obviously too large for crushing, which is not expedient, except for very small stones; it must, therefore, be cut out, and how is this to be done? In some respects there is, and long has been, a great approach to unanimity on this subject, but a point of the utmost importance, with reference to the safety of the operation, which is still not yet sufficiently understood or recognised by the profession generally. All in this country are agreed that it is not expedient to cut above the pubes, or to use the recto-vesical operation, and that the lateral operation of Cheselden is the best; all are also agreed that the incision in the skin should be free and large in proportion to the size of the parts, and that its anterior extremity should be situated at the raphe of the perinæum; also that any resisting fibres of the levator ani, or other perineal muscles, should be divided, and that the incision of the urethra should not extend further forwards than the middle of the membranous portion. So far, ninety-nine surgeons out of a hundred are agreed; but now comes the difficulty, how far must the incision be carried through the prostate? In order that you may understand this question fully, it will be necessary for us to begin at the beginning, and to take a rapid glance at the history of the subject. The great fundamental error in the operation of lithotomy formerly arose from the notion that incisions through membranous parts were mortal; as the neck of the bladder being a membranous part, it was supposed that to cut it would be to insure the death of the patient. This prejudice was the foundation of the operation by the "*apparatus major*," as it was called, in which an incision was made as far as the prostate, and then instruments were introduced, in order to dilate the prostatic part of the urethra and neck of the bladder. This, however, was a delusion, for the part admits of dilatation only to a small extent, and if stretched to a greater degree must be torn, so that the operation by the apparatus major was really a process of tearing, except when a very small stone required to be removed. The next step was the sudden and surprising appearance of Frere Jacques, who, throwing aside the details of the tedious process previously used, as John Bell expressed it, thrust his dagger-shaped knife into the patient's hip, with no other guide than a round staff, and extracted stones of the largest size without difficulty or delay. It is true his patients generally died, but some recovered, and this was enough to open the eyes of the profession, and to show that the dread entertained of cutting the neck of the bladder was groundless. The surgeons of France, Holland, and England proceeded to act upon this conviction; but certainly the most celebrated step in advance, at least as we think, was made by that distinguished countryman of ours, if I may so call him, Cheselden, who operated by his lateral method with a success that, taking all in all, has hardly been surpassed. It might be said that the operation was now perfect, and that nothing more was required. Unfortunately, however, the difficulty was only begun, the question being, what did Cheselden cut? You may say, there could be no difficulty in ascertaining this; as being a good anatomist, he must have been himself aware what parts he divided, and would communicate the facts to others. You find, however, that eminent commentators differ in their statements as to his incision, and you will have little difficulty in understanding how this happened when you consider that the part is not within reach of sight, so that the operator himself can alone have any knowledge where the knife is going, and even he can judge but imperfectly at a part beyond the reach of touch. Rau was a very successful lithomist; he is even said to have operated in 3000 cases without losing a patient, though that is hardly to be credited; but Rau would never tell any one what he cut. Albinus thought he knew from what he had seen as his assistant, and described as Rau's an operation which misled Cheselden, who, having been induced to try it in consequence of Rau's great success, found that he lost two-thirds of his patients, and therefore went back to his own method. It appeared, in fact, that successful lithotomists were lucky enough to get into a knack of making their incisions safely, and that having acquired this power they were able to employ it themselves, but were unable to communicate it with any certainty to others. This appeared very unsatisfactory, and at an early period of professional career I endeavored to ascertain with precision what it was that required to be cut. It was plain that the success of some surgeons and the failure of others could not be due to chance, but that there must be some reason for it; and I had not investigated long before I thought I saw this reason. The prostate is made up for the most part of a texture of no great firmness, and which does not much resent injury. You will see ample evidence of this if you examine the prostate of a person who has been long treated for retention of urine by an unskilful operator. You will probably find numerous false passages in the gland made by forcible thrusts of instruments, yet these tracks are soundly healed, and the patient has been none the worse for them. But it appears that there is one part of the prostate that is not thus tolerant of injury, and this is placed just at its base. If you take a chestnut, and run a bougie through it from the apex to the base, as I have done with the one before you, and hold the bougie as if it were introduced into the bladder, you will be able to form a tolerable idea of the form and position of the prostate gland. It is agreed by all that the incision through it should not be made directly downwards or directly to one side, but midway between these two directions. Now, at the base of the glaad, where it joins the neck of the bladder, there is a dense texture, forming a sort of ring round the urethra, represented pretty well in superficial extent by the *hilum* of the chestnut; it is thickest under the mucous membrane, whence it gradually tapers away. This part of the prostate seems endowed with an extraordinary degree of sensibility. If you pass a bougie in a patient who has never had it done before, you may introduce the instrument up to the prostate and a little way into it without producing more than an odd sensation, scarcely amounting to discomfort on the part of the patient; but when you push it on to the neck of the bladder, he perhaps faints and falls at your feet. When a patient affected with stone is going down stairs, he suffers to a very great degree, and this appears to be due to the stone being then thrown on this sensitive part: and in the same way great suffering is experienced when the calculus is forced upon the neck of the bladder in the empty and contracted condition of that organ. This sensitive part is, as I before remarked, of very dense texture, and it is very tough and unyielding. I have tried careful dilatation of it on the dead subject, but have hardly been able to enlarge the ring to more than a circle of an inch in diameter. There can be no doubt that if this texture be torn, the patient will die; if it be extensively lacerated, death will probably occur within two days; and if the injury be of less extent, chronic inflammation will set up about the neck of the bladder, leading with no less certainty to a fatal termination. If, however, the prostate be divided as far into its substance as this ring extends, the rest of the gland tears very readily, and without any bad consequence, the muscular fibres of the neck of the bladder separate from each other, and the mucous membrane also yields to the dilating force, so that sufficient space can be obtained without difficulty for the extraction of any stone which the outlet of the pelvis will admit. The result, then, of my investigation was that the different success of different surgeons must depend upon this ring being divided sufficiently by some, and not by others. I then inquired into the means used by different surgeons for making the incision in the prostate, and found that all those who were successful used instruments that might ensure the complete division of the texture in question. This odd-looking instrument is the knife used by the late Mr. George Bell, of this city, who operated very extensively and successfully for stone. This, again, is Dubois' knife, and this the "bistourie cachee," which, when properly set, will ensure, like the other two instruments, the necessary division of the prostate. The most curious illustration of this subject is furnished by the knife of M. Le Dran, who is said never to have lost a single case. It is a curious fact, that I at one time contrived an instrument of precisely similar form, and used it until the introduction of chloroform made it no longer necessary; and you may believe, that instead of being sorry to find that I had been anticipated by Le Dran, I was very glad to find the precedent of so distinguished an operator. The knife was run along a straight director introduced into the bladder through the wound in the perinæum; and the blade, which is two-thirds of an inch in breadth, is sharp only along its oblique anterior edge. I still believe that such an apparatus is the most certain means of dividing the prostate to the proper extent, when the part is withdrawn beyond the reach of the finger, as it may be when the patient is conscious. When the prostate has been divided sufficiently freely, and the part has been properly dilated with the finger, the urine necessarily escapes, and accordingly I never knew a successful operator for stone introduce the forceps till this had occurred. Not long ago, however, I read to my extreme surprise, a recommendation from a lecturer on surgery, to take advantage of the flow of urine, so that the stone might be washed into the grasp of the forceps. Now, I do not hesitate to say, that any surgeon who so conducts his operation that the urine does not escape till the forceps are introduced, cannot be a successful lithotomist; he may get on pretty well with young patients, but he will lose a large proportion of adults. Mr. Liston, one of the most successful operators for stone, would have been horrified at the idea of introducing the forceps before the urine escaped.

I had long taught the principle I am endeavoring to impress upon you, but had great difficulty in doing so, because Mr. Liston, whose authority was deservedly much respected, had adopted an unhappy way of describing, and a still more unhappy way of representing, the incision through the prostate. In the first three editions of his Practical Surgery, he represents a section of the prostate with an incision, leaving the base entire, and says—"the less that is cut the greater will be the patient's safety." you will remember, the question is not between recklessly extensive incisions, and very limited ones, but between the latter and incisions sufficiently free to insure the division of the unyielding ring. I had a correspondence with Mr. Liston upon this subject, in which I insisted on the mischievous tendency of the expressions he had used, whilst he,* on the other hand, maintained their correctness. At length, in the fourth edition, published only a year before his death, there appeared a paragraph, which I read with very great satisfaction, and which I will now read to you: "I introduce a sketch here originally published some time ago in illustration of some of my surgical lectures in the "Lancet," to show what it is indispensable to cut. If that is effected, the dilatation can be carried to any required extent. The two white triangular spaces at the base of the gland, in front and behind, show a section of the dense unyielding fibrous tissue into which the muscular fibres are inserted; this band effectually prevents dilatation on enlargement of the orifice of the bladder beyond a certain and very limited extent, without laceration, dreadful suffering, and imminent danger." This you see completely confirms what I had so long labored to establish. Entertaining this opinion very strongly, I confess I never felt anything more distressing than the consciousness that I had not cut what I ought to have cut, in consequence of my not being able to reach the part with my finger. When this happened the patient died; and yet the result was quoted as an argument against a free incision, while I well knew that the error had been of an opposite kind, and it was on this account that I introduced the knife which I have shown you; but which, I am happy to say, is no longer necessary, as the patient, when under chloroform, does not make those involuntary muscular efforts by which the parts were, in former times, withdrawn beyond the reach of the operator, unless he had a very long forefinger. If, on introducing your finger to dilate the deep part of the incision, you feel you have not cut enough, pass a straight probe-pointed bistoury along your finger, and, by a gentle sawing motion, carry the incision on the extent that you find necessary, in order to enable you to dilate with facility. There is only one other point to which I have time to allude at present, and that is the form of the forceps. It is a curious circumstance, that Cheselden, who, I do not hesitate to say, of all the surgeons of this country, has shewn the greatest surgical genius as an operator, has left no work on surgery, except some remarks and figures of instruments appended to Le Dran's work on the operations in surgery, translated by another man. It is this work which I now hold in my hand; here you see represented, in one of the plates, the operation of amputation by double incision to which I alluded in a late lecture; here is a portrait of his lithotomy forceps, and so accurate was everything he did, that forceps can now be constructed by an instrument maker from this pattern. Observe the difference between these forceps which I had constructed many years ago, after Cheselden's pattern, and this pair, which I obtained from an instrument shop; the blades of the former are, you observe, considerably longer than those of the other pair, and, therefore, approach nearer to a state of parallelism when the stone is seized, and the extraction is thus rendered much more easy. I may remark, that you will generally see the best operators slow in making their incisions, and rapid in extracting the stone. The incisions require to be made with precision, and dilatation must be thoroughly effected with the finger; and it is no matter how long these parts of the operation occupy; but when they have been properly done, the extraction of the stone is a comparatively easy procress.

[Mr. Syme performed the operation on the 3rd of February. Having introduced the staff, which was a small one, on account of the presence of the stricture, and made the incisions in the usual way, he found that the prostate was very tough, and did not yield readily to dilatation by the finger, and therefore increased the incision in the prostate with a straight probe-pointed bistoury. The stone was now readily extracted, and measured an inch and three-quarters in length, an inch and a half in breadth, and one inch in thickness, and appeared to be phosphatic. A gum-elastic tube was introduced into the bladder through the wound, and retained forty-eight hours; little blood was lost at the operation, and no hemorrhage occurred after it. The urine which came by the tube presented a remarkable contrast to that which had been passed before the operation, being clear and free from albumen, instead of thick and highly albuminous. The patient was also freed, by the operation, from the pain, he had previously endured; but his tongue continued for a long time to have the same dry character as before, he was slow in regaining his appetite, and was a good deal fretted by the stricture, which prevented the urine resuming its natural channel, and refused to yield to dilatation. This obstinacy of the stricture appeared to be accounted for by its duration, which had not been previously fully known, the wife of the patient stating that he had been for sixteen years in the habit of drawing off his urine by catheter; and it therefore appeared that, instead of the stone being the cause of the stricture, as had been at first supposed, the contraction of the canal had given rise to the calculus, the size of which was hardly consistent with so long a period of duration. At length, seven weeks after the operation, the wound was so very nearly closed that the patient became unable to pass his urine, which it was necessary to draw off two or three times a day by a No. 3 catheter, which was the largest instrument that could be passed through the tight contraction at the bulb. Mr. Syme, therefore, determined to divide the stricture, the patient being in other respects in good health, though still weak, and performed the operation on the 26th of March at a clinical lecture. The proceeding presented no remarkable feature, and a No. 10 catheter, provided with a stop-cock, was tied in the bladder as usual. It was removed at the end of forty-eight hours, after which the urine came for some time by the wound, but gradually resumed its natural passage, full-sized bougies being passed occasionally, on account of a tendency to contraction near the orifice. The lithotomy wound soon closed completely, while that of the operation for stricture remained open for a considerable time, the urine also coming away involuntarily. Latterly, however, he has acquired considerable command over his urine, and now (April 28th) can retain it as long as four hours at a time, and then passes it with a voluntary effort. All tendency to contraction of the urethra has now disappeared, and it admits a No. 11 bougie with perfect facility. The patient is also rapidly improving in general health, and is stronger than for many years before the operation, while his urine is quite clear and free from albumen.

On the 19th March, a man, twenty-nine years old, from Glasgow, was admitted into the hospital, complaining of very frequent micturition, and pain in the penis during the act and some time after it; and occasionally passing blood. These symptoms had existed for a year and a quarter, and soon after their commencement he began to suffer from abscess in the perinæum which resulted in fistulous openings beside the anus, through which urine passed, and he was admitted, with this state of things, into the Glasgow Infirmary, where he remained four months, during which time the fistulous apertures were freely dilated, and the scars of the incisions were to be seen at each side, extending from about the tuberosity of the ischium into the anus. The fistulous openings, however, did not close completely, and he continued to suffer from urinary symptoms. On his admission, Mr. Syme sounded him and detected a calculus, and on the 28th of March, performed lithotomy. The induration of the perinæum, which had occurred in connexion with the abscesses, made the first part of the incisions unusually difficult; the rest of the operation was easily accomplished, the stone being about the size of a small walnut. In the course of the afternoon some hemorrhage occurred, which was found to proceed from the transversalis perinæi artery, and ceased immediately when it was tied. The patient afterwards went on in every respect favorably; on the removal of the tube, forty-eight hours after the operation, the urine came at once partly by the natural passage, and the quantity taking this course increased, till, on the twenty-fourth of April, the urine ceased to come by the wound. The fistulous openings are also now (April 28th) soundly healed. His general health is also good.

On the 2nd of April a young man, twenty-four years old, presented himself at the Infirmary, complaining of urinary symptoms, from which he had suffered from childhood, the most prominent being frequent micturition, and pain at the close of the act, and lasting for some time after it. A small stone was discovered on examination of the bladder, and Mr. Syme, who saw him next day, pronounced it a mulberry calculus, from the sensation it communicated when struck by the sound. On the following day Mr. Syme performed lithotomy, the operation presenting no difficulty, and the calculus proving to be of oxalate of lime, as had been suspected, with very deep intervals between the pointed arborescent processes which formed its surface. The patient has done well on the whole, some little bleeding after the removal of the tube, forty-eight hours from the operation, being the only circumstance to interfere with his recovery, and this appeared due rather to excess of blood in the system than to any serious cause, and ceased when the tube was re-introduced, so as to prevent the straining on micturition which previously occurred. At the present time (April 28th) the urine comes almost entirely by the natural passage, and his general health is good.]

